# Supplementary material for: Involvement of N-glycan in Multiple Receptor Tyrosine Kinases Targeted by Ling-Zhi-8 for Suppressing HCC413 Tumor Progression
Source: Cancers (Basel). 2018 Dec 21;11(1):9. doi: 10.3390/cancers11010009 (PMC6356446; doi:10.3390/cancers11010009)
Supplement: Supplementary file 1 [file cancers-11-00009-s001.pdf]

cancers

Supplementary Material

Involvement of N-glycan in Multiple Receptor Tyrosine Kinases Targeted by Ling-Zhi-8 for Suppressing HCC413 Tumor Progression

Ren-In You, Wen-Sheng Wu, Chuan-Chu Cheng, Jia-Ru Wu, Siou-Mei Pan, Chi-Wen Chen and Chi-Tan Hu

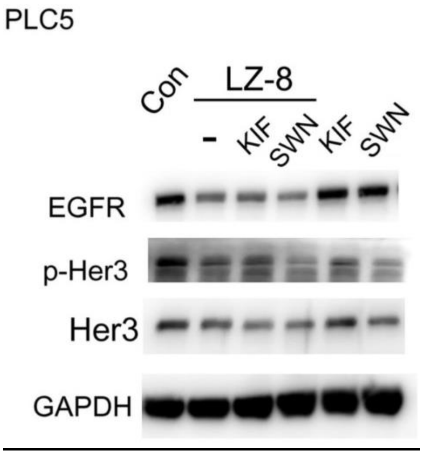

(**A**)

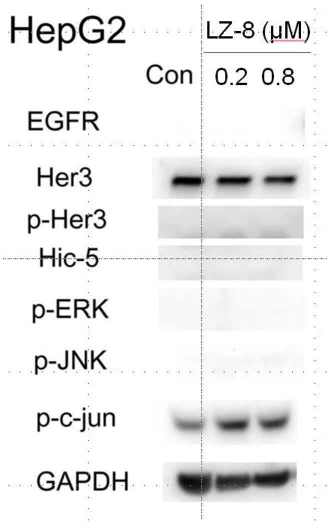

(**B**)

Figure S1. LZ-8 suppressed expression and phosphorylation of RTK in PLC-5 but not HepG2 cells. PLC-5 (**A**), HepG2 (**B**) cells were treated with LZ-8 at indicated concentration (**B**) or pre-incubated with the indicated mannosidase inhibitor for 16 h, followed by treatment with LZ-8 for 24 h (**A**). Western blot of indicated molecules were performed. GAPDH was used as internal control. In (**A**), KIF: Kifunensine (10 μM), SWN: Swainsonine (5.8 μM).

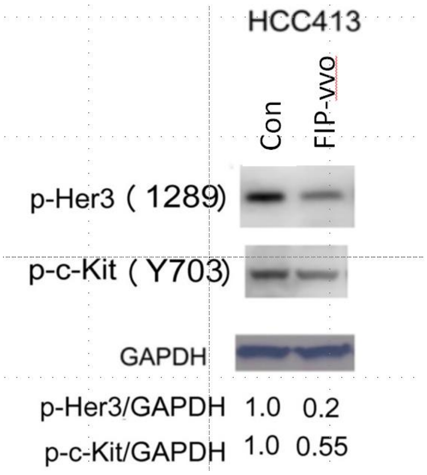

Figure S2. FIP-vvo suppressed phosphorylation of Her3 and c-Kit in HCC413. HCC413 cells were treated with FIP-vvo (0.4 μM) for 24 h, Western blot of indicated molecules were performed. GAPDH was used as internal control. The number indicated below is the relative ratio of intensity of indicated molecules vs GAPDH, taking the control as 1.0.

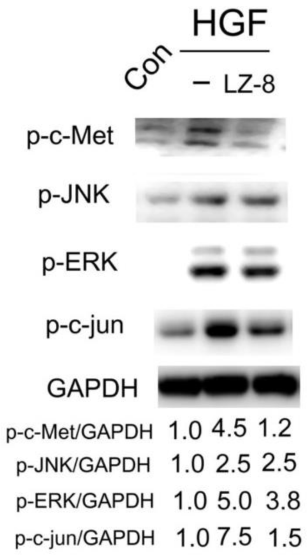

(**A**)

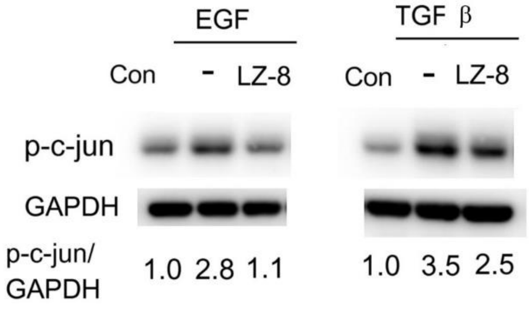

(**B**)

Figure S3. LZ-8 suppressed growth factor induced MAPK signaling in HCC340. HCC340 were untreated (Con), treated with indicated growth factor alone or growth factor coupled with LZ-8 (**A**, **B**). Western blot of indicated molecules were performed using GAPDH as internal control. The numbers below each figure are the ratio of indicated molecules *vs* GAPDH. The data were average of three reproducible experiments with C.V. of 8.0.

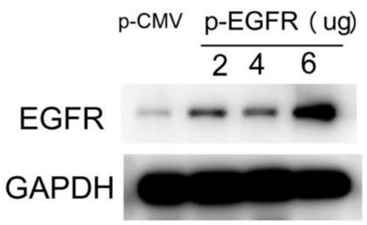

Figure S4. Elevation of EGFR in HCC340 transfected with EGFR-expressing plasmids. HCC340 cells were transfected with different amounts of EGFR expressing plasmid for 48 h, followed by Western blot of EGFR, using GAPDH as an internal control.

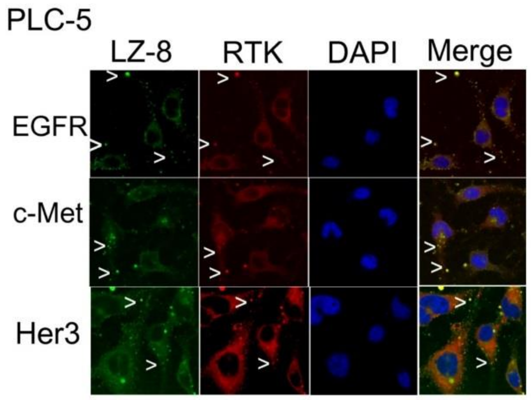

(**A**)

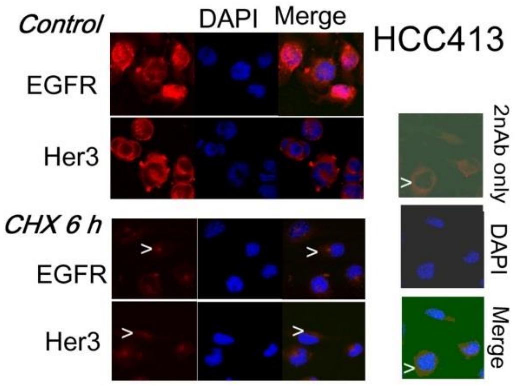

(**B**)

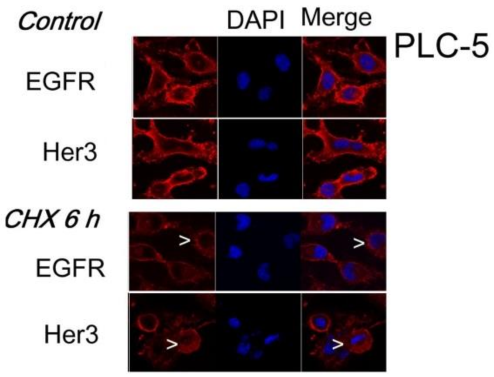

(**C**)

Figure S5. Binding of LZ-8 with RTKs in PLC-5, and protein synthesis inhibitor reduced intracellular stainings of RTK in HCC413 and PLC-5. PLC-5 (**A**, **C**) and HCC413 (**B**) were treated with FITC labelled LZ-8 for 5 min (**A**) or untreated (control) or treated with 10 μM cycloheximide (CHX) for 6 h (**B**). Immunostainings for indicated RTKs were performed, followed by confocal fluorescence analysis. In (**A**), the white arrow heads indicated the colocalization of LZ-8 with indicated RTKs which were revealed as yellow dots in the Merge image (resulted from overlapping of green and red colors of LZ-

8 FITC and indicated RTK, respectively). In (**C**) and left panel of (**B**), the white arrow heads indicated the perinuclear staining of indicated RTK. In the right panel of (**B**), the cells were stained only by rhodamine-labelled 2nd Ab commonly used for RTK immunostaing. The white arrow heads indicated the perinuclear background staining. DAPI was included as nuclear counter staining. The data were representatives of two reproducible experiments.

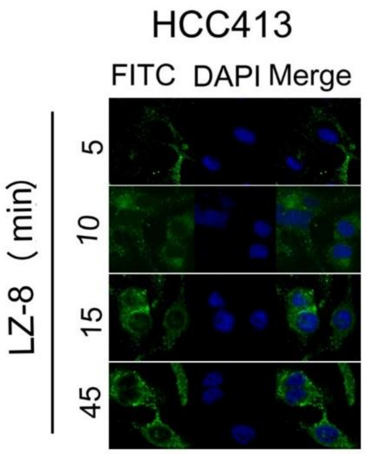

(**A**)

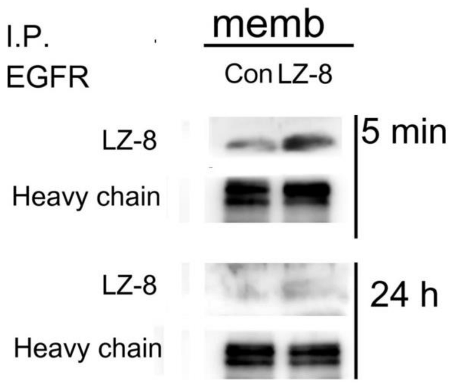

(**B**)

Figure S6. Time dependent internalization of LZ-8 and differential subcellular binding of LZ-8 with EGFR in HCC413. HCC413 cells were treated with LZ-8 (0.2 μM) for the time indicated (**A** and **B**). In (**A**) after fixation, immunostaining for LZ-8 were performed using FITC conjugated 2nd Ab, followed by confocal fluorescence analysis. In (**B**) membrane fractions of HCC413 were used for IP EGFR and Western blot of LZ-8, using Ab heavy chain as internal control. The data were representatives of two reproducible experiments.

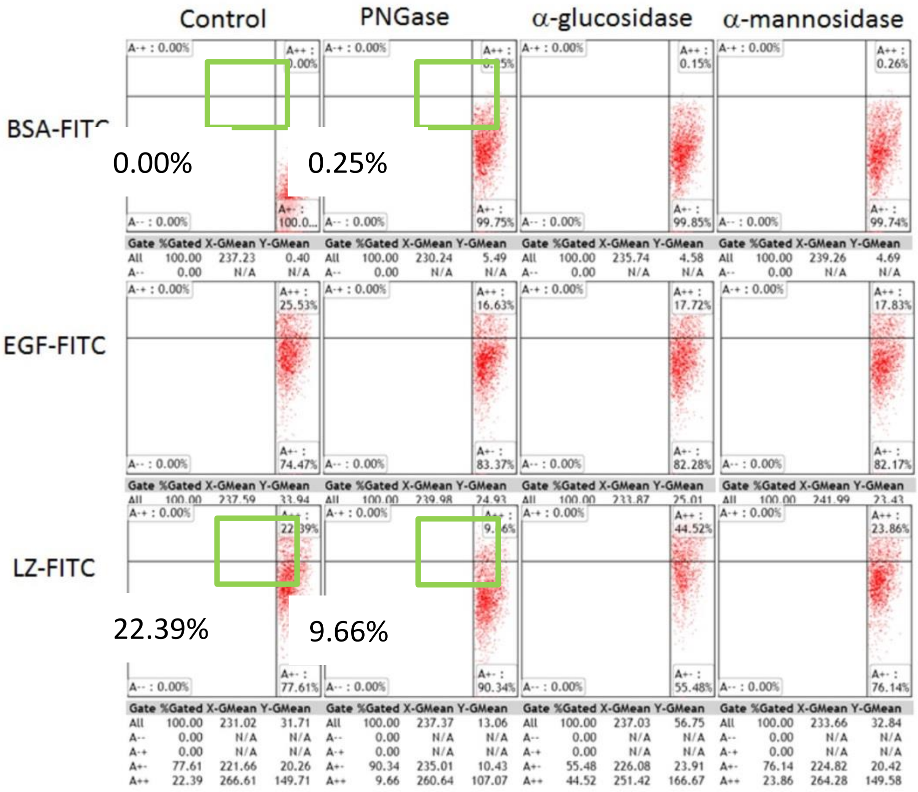

(**A**)

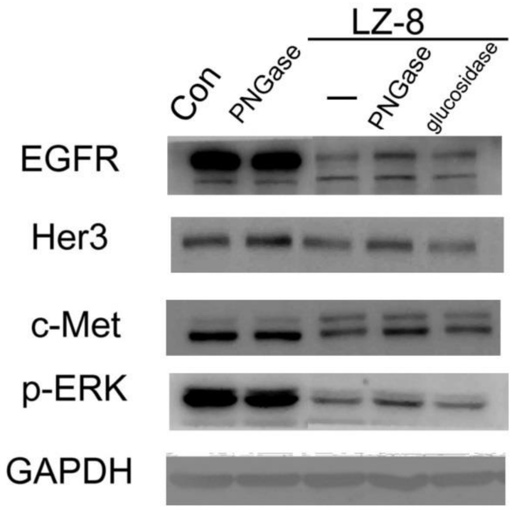

(**B**)

Figure S7. PNGase rescued the LZ-8-inhibited RTK expression and signal transduction in HCC413. HCC 413 cells were pretreated with PNGase (100 U/mL), α-glucosidase (100 U/mL) (**A**, **B**), mannosidase (100 U/mL) (**A**) for 4 h, followed by treatment with LZ-8-FITC for 15 min (**A**) or LZ-8 for 24 h (**B**). Flow cytometry (**A**) and Western blot of indicated molecules, using GAPDH as a control (**B**), were performed. In (**A**), the green rectangle indicated the G-mean (%) of FITC fluorescence in the BSA-FITC control and the LZ8-FITC group with or without PNGase pretreatment. The numbers of indicated G-means are enlarged in each panel. In (**B**), the data is representative of two reproducible results.

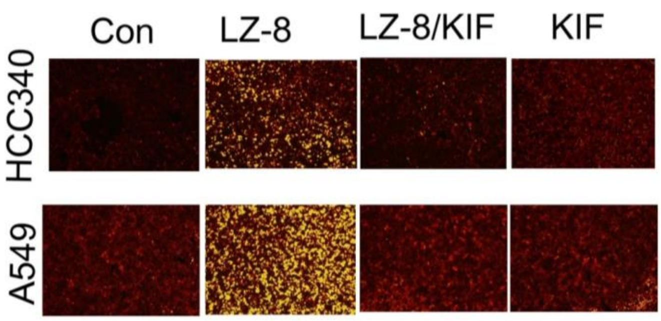

(**A**)

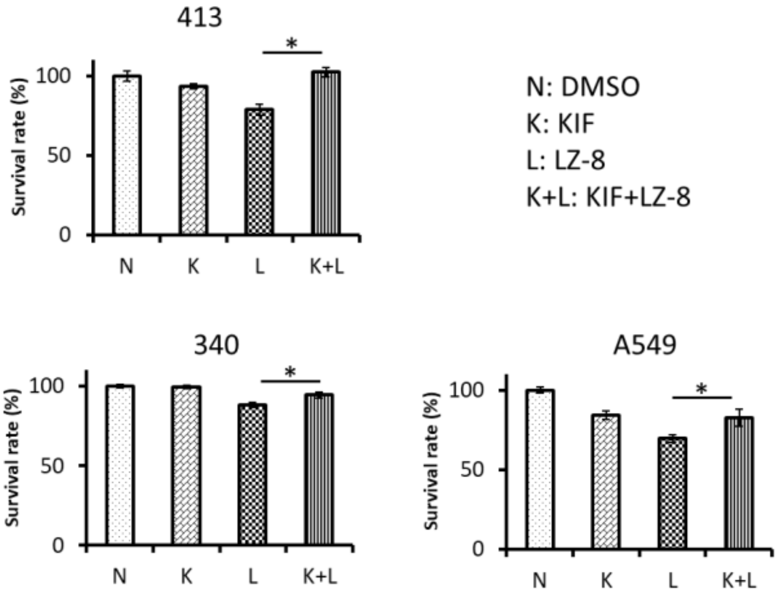

(**B**)

Figure S8. KIF prevented LZ-8-inhiited cell migration and growth of various cancer cell lines. The indicated cell lines were untreated (Con), treated with LZ-8, LZ-8 coupled with KIF or KIF alone. Transwell migration assay (**A**) and MTT assay (**B**) were performed. In (**A**), pictures were taken 24h after indicated treatment under phase contrast microscope (100X magnifications). In (**B**), (*) represents the statistically significant difference between LZ-8 (L) and the KIF+LZ-8 (K+L) group ($p < 0.005$, n = 3).

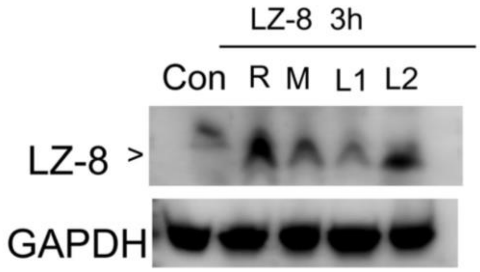

Figure S9. Distribution of LZ-8 within SCID mice liver after intraperitoneal injection. Mice were untreated or treated with LZ-8 (40 μg/mice) by intraperitoneal injection for 3 h. Then the mice were sacrificed for obtaining the liver tissues to examine the distribution of LZ-8. Two mm^3 of tissue slices were cut from each of the right (R), middle (M) and left (L1 and L2) liver lobe. Tissue lysates were extracted for Western blot of LZ-8 using GAPDH as an internal control. The arrow head indicates the position of LZ-8 (12 kDa). The data was representative of 2 reproducible results.
